# Tumor Necrosis Factor (TNF) Receptor Expression Determines Keratinocyte Fate upon Stimulation with TNF-Like Weak Inducer of Apoptosis

**DOI:** 10.1155/2019/2945083

**Published:** 2019-12-05

**Authors:** Xuening Wang, Dan Cheng, Guanglei Hu, Lili Liang, Fei Tan, Tong Xiao, Shengxiang Xiao, Yumin Xia

**Affiliations:** ^1^Department of Dermatology, The Second Affiliated Hospital, School of Medicine, Xi'an Jiaotong University, Xi'an, China; ^2^College of Life Sciences, Wuhan University, Wuhan, China

## Abstract

The interaction between tumor necrosis factor- (TNF-) like weak inducer of apoptosis (TWEAK) and fibroblast growth factor-inducible 14 (Fn14) regulates the fate of keratinocytes, depending on the relative expression of TNF receptor (TNFR) 1 or TNFR2. However, the precise mechanism underlying this TWEAK-mediated regulation remains unclear. The aim of this study was to provide comprehensive insight into the roles of Fn14, TNFR1/2, and other relevant molecules in the fate of keratinocytes. Further, we sought to elucidate the structural basis for the interaction of TWEAK and Fn14 in regulating cellular outcomes. Normal keratinocytes (mainly expressing TNFR1) and TNFR2-overexpressing keratinocytes were stimulated with TWEAK. Through immunoprecipitation and Western blotting of keratinocyte lysates, we elucidated the associations between Fn14, TNFR-associated factor 2 (TRAF2), cellular inhibitor of apoptosis protein 1 (cIAP1), and TNFR1/2 molecules. Additionally, we found that TRAF2 exhibited binding to Fn14, cIAP1, and TNFR1/2. Our data suggest that TWEAK induces apoptosis in normal keratinocytes and proliferation in TNFR2-overexpressing keratinocytes in a TNF-*α*-independent manner; however, inhibition of TRAF2 appears to reverse this effect. Interestingly, the interaction between TWEAK and Fn14 increased TNFR1-associated death domain protein and caspase-8 expression in normal keratinocytes and promoted cytoplasmic import of cIAP1 in TNFR2-overexpressing keratinocytes. In conclusion, we found that the Fn14-TRAF2-TNFR signaling axis mediates TWEAK's regulation of the fate of keratinocytes, possibly in a manner involving the TNF-*α*-independent TNFR signal transduction.

## 1. Introduction

Recent studies have demonstrated that the tumor necrosis factor- (TNF-) related weak inducer of apoptosis (TWEAK)/fibroblast growth factor-inducible 14 (Fn14) signaling pathway plays an important role in the pathogenesis of various skin diseases including malignancies [1], human papillomavirus (HPV) infection [2], lupus erythematosus [3, 4], bullous pemphigoid [5], psoriasis [6–8], burn wounds [9], and other inflammatory diseases [10, 11]. Although some of these conditions are associated with excessive proliferation of epidermal keratinocytes, others exhibit increased apoptosis. TWEAK functions by binding to its sole receptor Fn14, which is expressed by keratinocytes in general and overexpressed in tumorigenic and inflammatory conditions [1, 2, 12]. Activation of TWEAK/Fn14 not only triggers the production of proinflammatory cytokines such as regulated upon activation normal T cell expressed and secreted (RANTES) but also regulates the fate of keratinocytes depending on local microenvironments [2, 3, 13].

Herein, we found that the expression profile of TNF receptors (TNFR) largely determines the effect of TWEAK/Fn14 activation on keratinocytes *in vitro* [12]. A relative abundance of TNFR1 expression in keratinocytes is associated with apoptosis upon TWEAK activation [4]. Conversely, TNFR2 upregulation enhances the proliferation of keratinocytes upon TWEAK stimulation [1, 2, 8, 13]. These findings were consistent with histological observations. Skin lesions of lupus erythematosus characteristically have apoptotic keratinocytes, which are more readily observed in ultraviolet B-radiated skin [3]. Interestingly, ultraviolet B radiation promotes the expression of both Fn14 and TNFR1 in keratinocytes [4]. On the other hand, HPV type 16 and psoriatic cytokines lead to increased expression of Fn14 and TNFR2 in keratinocytes [2, 13]. Moreover, exogenous TWEAK can regulate the cell cycle of keratinocytes in a manner independent of TNF-*α* [2, 13, 14]. Therefore, the specific pattern of TNFR expression appears to play a significant role in determining the effect of TWEAK on keratinocytes.

However, specific connections between Fn14 and TNFR molecules have not yet been elucidated. It has previously been shown that upon binding to TWEAK, Fn14 can recruit a cellular inhibitor of apoptosis protein 1- (cIAP1-) TNFR-associated factor 2 (TRAF2) complex and sensitize tumor cells to TNF-*α* [15]. We have also previously demonstrated that HPV16 E6/E7 transfection increases the expression of TRAF2, and TRAF2 inhibition further leads to a reduction in TWEAK-facilitated proliferation in these keratinocytes [2]. These findings strongly suggest that TRAF2 mediates the HPV16 infection-induced switch from apoptotic to proliferative outcomes in keratinocytes as a result of the TWEAK/Fn14 interaction. It has also been shown that TRAF2 can bind to TNFR, which recruits TRAF2 when stimulated by TNF-*α*, thereby triggering downstream signals [16]. Hence, we speculate that an Fn14-TRAF2-TNFR signaling axis may mediate the regulation of keratinocytes' cellular responses to TWEAK/Fn14.

This study is aimed at revealing the exact interactions between Fn14, TRAF2, and TNFR molecules and at elucidating the structural basis for the TWEAK/Fn14-mediated regulation of diverse fates in keratinocytes.

## 2. Materials and Methods

### 2.1. Cell Culture and siRNA Transfection

Human primary keratinocytes and EpiLife® medium (supplemented as described in [Supplementary-material supplementary-material-1]) were purchased from Life Technologies Company (Carlsbad, CA). Cells were routinely starved for 24 h, and then, the media were supplemented with recombinant TWEAK (100 ng/ml; R&D Systems, Minneapolis, MN) or TNF-*α* (10 ng/ml; R&D Systems) for 48 h. This duration and concentration of TWEAK and TNF-*α* have been shown to be efficient for inducing the changes of cell fate of normal keratinocytes [4, 13, 17]. Some cells were simultaneously treated with an IAP inhibitor Birinapant (1 *μ*M; Selleck Chemicals, Houston, TX). To exclude the potential effect of TWEAK on autocrine TNF-*α* production, the TWEAK-alone-stimulated keratinocytes were further analyzed. We verified that TNF-*α* was not detectable in supernatants while its mRNA was upregulated in cells. However, the regulation of TNF-*α* mRNA levels is irrelevant (Supplementary Fig. [Supplementary-material supplementary-material-1]).

In some experiments, keratinocytes were pretransfected with Fn14 (Cat # AM16708; Life Technologies), TRAF2 (Cat # 4392420; Life Technologies), or control (Cat # AM4611; Life Technologies) siRNA before TWEAK or TNF-*α* stimulation. As reported previously [5], the mixtures of siRNA oligonucleotides and Lipofectamine 2000 transfection reagent (75 pmol siRNA: 7.5 *μ*l of reagent; Life Technologies) were added to cell cultures in six-well plates for 24 h. To exclude the effect of siRNA on innate immune responses, TNF-*α*, RANTES, and interferon *γ* were analyzed accordingly and were both found to be undetectable in the supernatants (data not shown) and have unchanged mRNA levels in cells after siRNA transfection (Supplementary Fig. [Supplementary-material supplementary-material-1]). The transfection efficiencies were verified by quantitative real-time PCR (qRT-PCR) and Western blotting and were found to be >80% (Supplementary Fig. [Supplementary-material supplementary-material-1]).

### 2.2. Retroviral Infection

As described previously [18], retroviral infection was initiated when keratinocytes were under logarithmic growth phase. In summary, the pBabe vector (Cell Biolabs, San Diego, CA) with an inserted TNFR2 gene was used for infection after high-titer retroviral supernatants were prepared. After infection, cells were selected using G418 solution. The efficiency of TNFR2 expression was verified by both qRT-PCR and Western blotting (Supplementary Fig. [Supplementary-material supplementary-material-1]).

### 2.3. qRT-PCR

Total RNA was extracted from cell cultures using the PureLink RNA kit (Life Technologies). Reverse transcription was performed using a commercial cDNA kit (Applied Biosystems, Carlsbad, CA). qRT-PCR was carried out on the 7900HT Fast PCR system (Applied Biosystems). SYBR Green Master Mix (Life Technologies) was used as the fluorescent dye. The sequences of the primers used (Jieqing Biotech, Wuhan, China) are shown in Supplementary [Supplementary-material supplementary-material-1] online. The expression levels of the objective genes were calculated by using the 2^−*Δ*Ct^ method.

### 2.4. Flow Cytometry

An apoptosis assay was performed by flow cytometry [4]. Keratinocytes were harvested and resuspended in annexin binding buffer. Annexin V-Alexa Fluor 488 (Life Technologies) and 7-aminoactinomycin D (BD Biosciences, San Jose, CA) were added to these samples. Flow cytometry was performed on an LSR II Flow Cytometer (BD Biosciences). Compensation and analysis of the data were carried out using FlowJo software (TreeStar Inc., Ashland, OR). In this assay, late apoptotic cells were defined as positive for both annexin and 7-aminoactinomycin D.

### 2.5. Proliferation Assay

Cell proliferation was determined with CellTiter 96® Solution (Promega Corporation, Madison, WI). Absorbance at 490 nm was recorded using a 96-well plate reader (Thermo Fisher Scientific, Waltham, MA). The values from each plate were normalized to that of the blank controls.

### 2.6. Immunofluorescence

Immunofluorescence was performed as previously described [19]. Briefly, keratinocytes were grown on a glass-bottom culture dish (MatTek Corporation, Ashland, MA) and fixed in 4% paraformaldehyde solution. Rabbit IgG targeting cIAP1 (clone # ab108361) or TRADD (clone # ab110644) and Alexa Fluor 488-labeled rabbit anticaspase-8 IgG (clone # ab206068) were used as primary antibodies (2 *μ*g/ml; Abcam, Cambridge, MA). Alexa Fluor 488-labeled goat anti-rabbit IgG (2 *μ*g/ml; clone # ab150077; Abcam) was used as a secondary antibody. Cells were counterstained with 4′,6-diamidino-2-phenylindole (DAPI) before observation under a digital confocal microscope (Leica Co, Wetzlar, Germany).

### 2.7. Immunoprecipitation and Western Blotting

Protein extracts were prepared using RIPA lysis buffer supplemented with a protease inhibitor cocktail (Life Technologies). In some experiments, separate cytoplasmic and nuclear protein fractions were extracted using the NE-PER™ Extraction Reagents (Thermo Fisher Scientific). MutS protein homolog 2 (nuclear) and heat shock protein 90 (cytoplasmic) were measured in the cytoplasmic and nuclear fractions, confirming no cross contamination (Supplementary Fig. [Supplementary-material supplementary-material-1]) [20].

Immunoprecipitation was performed using a protein G kit (Roche, Indianapolis, IN) [21]. Lysates were mixed with target IgG (or control IgG) (Abcam), followed by overnight incubation at 4°C. Protein G suspension was then added to the mixtures. After centrifuging, the supernatants were discarded while the pellets were resuspended for Western blotting.

Protein samples were separated on electrophoresis gels and then transferred onto polyvinylidene difluoride membrane (Millipore, Billerica, MA). Rabbit IgG targeting Fn14 (clone # ab109365), TNFR1 (clone # ab68160), TNFR2 (clone # ab109322), cIAP1 (clone # ab108361), TRAF2 (clone # ab126758), RIP1 (clone # ab125072), TRADD (clone # ab110644), caspase-3 (clone # ab32351), caspase-8 (clone # ab32397), MutS protein homolog 2 (clone # ab227941), heat shock protein 90 (clone # ab109704), or *β*-actin (clone # ab124964) was used as the primary antibody (1 *μ*g/ml; Abcam). Horseradish peroxidase-labeled goat anti-rabbit IgG (0.5 *μ*g/ml; ab6721; Abcam) was used as the secondary antibody. Signal was developed using a commercial ECL kit (Millipore). The specificity of anti-cIAP1 IgG was validated by immunoprecipitation and mass spectrometry (Supplementary Fig. [Supplementary-material supplementary-material-1]). Band intensities were quantified using ImageJ software (National Institutes of Health, Bethesda, MD).

For the detection of ubiquitinated RIP1, denatured lysates were subjected to immunoprecipitation with rabbit anti-RIP1 IgG (clone # ab125072; Abcam) and probed with antiubiquitin IgG (1 *μ*g/ml; clone # ab134953; Abcam) to detect the incorporation of endogenous ubiquitin [22].

### 2.8. Surface Plasmon Resonance (SPR) Analysis

The affinities between different protein molecules were determined on a Biacore 3000 instrument (Biacore, Piscataway, NJ) [23]. Recombinant human cIAP1 (31 kDa), Fn14 (21 kDa), and TRAF2 (34 kDa) were purchased from Sigma-Aldrich Company (St. Louis, MO). Recombinant human TNFR1 (18.3 kDa) and TNFR2 (18.9 kDa) were purchased from Thermo Fisher Scientific. The proteins cIAP1, Fn14, and TRAF2 were prepared in 2-(N-morpholino)ethanesulfonic acid buffer and then immobilized to sensor chips (GE Healthcare, Port Washington, NY) at a concentration of 10 nM (Supplementary [Supplementary-material supplementary-material-1]). Samples were titrated from 0 to 100 nM in running buffer (pH 7.4, 20 mM KPO_4_, 130 mM KCl, 3.4 mM EDTA, and 0.005% Tween 20) and then injected to flow over the chip at a rate of 30 *μ*l/min. The simple Langmuir model (*A* + *B*⟷*AB*) was used to calculate binding kinetics. The association (*K*_a_) and dissociation (*K*_d_) values were calculated using Biacore evaluation software version 1.1.

### 2.9. Statistical Analysis

All data were expressed as mean ± standard deviation (SD). Statistical analysis was performed using Prism version 6.0 (GraphPad, La Jolla, CA). Analysis of variance (ANOVA) was used for comparisons among more than two groups, followed by a post hoc two-tailed Student's *t*-test between individual groups. The differences were considered significant at *p* < 0.05.

## 3. Results

### 3.1. Fn14/TRAF2/cIAP1/TNFR Form a Complex in Keratinocytes

**Figure 1:**
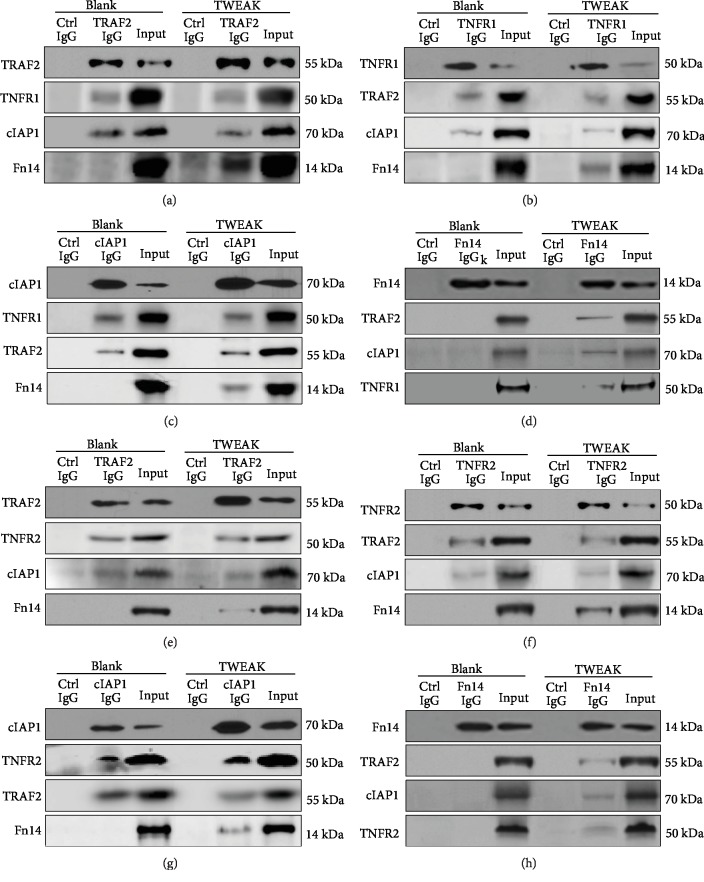
Detection of Fn14/TRAF2/cIAP1/TNFR complex in keratinocytes. Human primary keratinocytes were cultured *in vitro*, followed by extraction of protein lysates. Some cells were pretreated with TWEAK (100 ng/ml, 24 h). Using immunoprecipitation and Western blotting, we detected associations between the different potential binding partners. Proteins were precipitated by anti-TRAF2 (a), TNFR1 (b), cIAP1 (c), or Fn14 (d) IgG and then detected by other antibodies. Similarly, proteins were extracted from TNFR2-overexpressing keratinocytes and precipitated with anti-TRAF2 (e), TNFR2 (f), cIAP1 (g), or Fn14 (h) IgG. Input refers to the cell lysate that was aspirated before the addition of immunoprecipitation IgG. In contrast, control IgG refers to an antibody that is not related to the target protein. Data were obtained from five independent experiments, and none of the blots was stripped and reprobed. Representative images are shown.

By immunoprecipitation and Western blotting, the associations between Fn14, TRAF2, cIAP1, and TNFR1 molecules were detected in protein lysates generated from primary human keratinocytes. We found that anti-TRAF2 IgG precipitated both TNFR1 and cIAP1 proteins in both nonstimulated and TWEAK-stimulated keratinocytes (Figure 1(a)). However, anti-TRAF2 IgG only precipitated Fn14 protein in TWEAK-stimulated cells (Figure 1(a)). Similar results were observed when either the anti-TNFR1 or anti-cIAP1 IgG was used for immunoprecipitation (Figures 1(b) and 1(c)). Furthermore, anti-Fn14 IgG did not precipitate any of the other three proteins in nonstimulated keratinocytes but precipitated all of them in TWEAK-stimulated cells (Figure 1(d)).

TNFR2 is very weakly expressed in normal keratinocytes [2]. We found that TWEAK alone had no impact on TNFR expression in primary keratinocytes (Supplementary Fig. [Supplementary-material supplementary-material-1]). Thus, we constructed TNFR2-overexpressing keratinocytes using a retroviral vector. The associations between Fn14, TRAF2, cIAP1, and TNFR2 molecules were detected in the lysates of these keratinocytes, which were stimulated with TWEAK or control buffer. We found that the use of anti-TRAF2 IgG, anti-TNFR1, or anti-cIAP1 IgG precipitated the other proteins (except Fn14 in nonstimulated cells). However, anti-Fn14 IgG only precipitated the other proteins in TWEAK-stimulated cells (Figures 1(e)–1(h)).

### 3.2. TRAF2 Binds Specifically to Fn14 or TNFR

**Figure 2:**
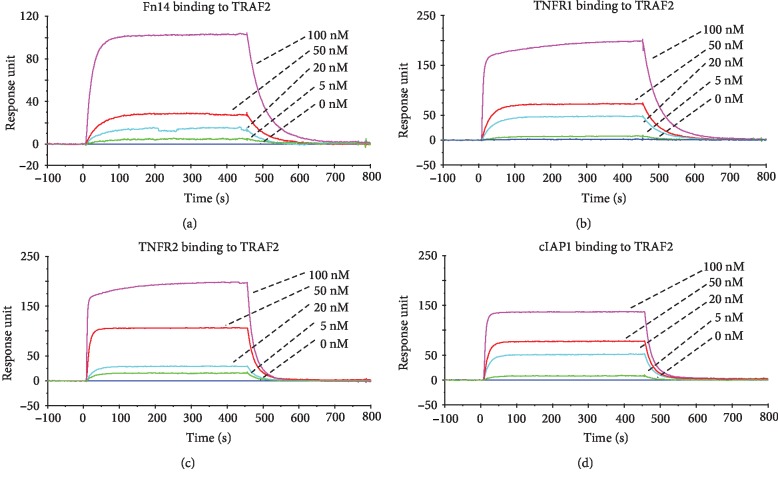
The specific binding of Fn14, TNFR1, or cIAP1 to TRAF2 molecule. These recombinant proteins were analyzed by SPR. TRAF2 protein was immobilized to a sensor chip, followed by running Fn14 (a), TNFR1 (b), TNFR2 (c), or cIAP1 (d) protein at a concentration rage of 0–100 nM. Data were obtained from five independent experiments. Representative images are shown.

The binding affinities of the interactions between Fn14, TRAF2, cIAP1, and TNFR1 (or TNFR2) molecules were determined by SPR. The bindings of Fn14, TNFR1, and TNFR2 to TRAF2 were characterized by high binding affinities (Figures 2(a)–2(c), Supplementary Fig. [Supplementary-material supplementary-material-1]). We also found that cIAP1 bound to TRAF2 (Figure 2(d)). The *K*_D_ values between these molecules are listed in [Supplementary-material supplementary-material-1]. There was no binding between Fn14 and cIAP1 (or TNFR1, TNFR2) or between cIAP1 and TNFR1 (or TNFR2) (Supplementary Fig. [Supplementary-material supplementary-material-1]).

### 3.3. TNFR Expression Profile Determines the Cell Fate of Keratinocytes

**Figure 3:**
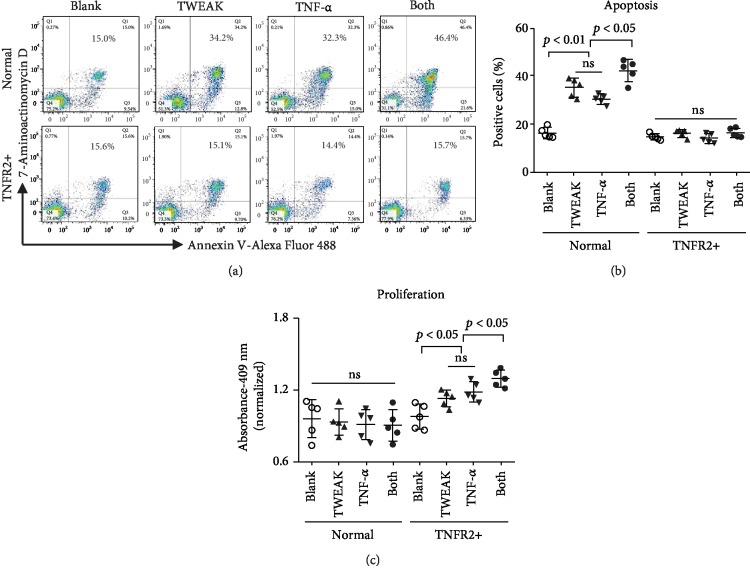
The effect of the TNFR expression profile on TWEAK- or TNF-*α*-induced cell fate in keratinocytes. Human primary keratinocytes were cultured *in vitro* and received 48 h stimulation with TWEAK (100 ng/ml) or TNF-*α* (10 ng/ml). TNFR2-overexpressing keratinocytes were constructed using a retroviral vector. (a) Apoptotic cells were quantified by flow cytometry. (b) The ratios of apoptotic cells were compared between different groups. (c) The proliferation of keratinocytes was quantitated by spectrophotometry, followed by comparison across groups. Data were obtained from five independent experiments. Representative images are shown. ns: not significant.

The effect of TWEAK on proliferation and apoptosis was studied in keratinocytes that predominantly expressed either TNFR1 or TNFR2. These experiments suggested that, administered alone, TWEAK increased the rate of apoptosis of normal keratinocytes (Figures 3(a) and 3(b)). Moreover, the combination of TWEAK and TNF-*α* exhibited an additive effect on the induction of apoptosis in normal keratinocytes (Figures 3(a) and 3(b)). Conversely, in TNFR2-overexpressing keratinocytes, the administration of TWEAK alone enhanced proliferation. Interestingly, the addition of TNF-*α* increased this proliferative effect (Figure 3(c)). We observed no obvious effect of TWEAK on the proliferation of normal keratinocytes or on the apoptosis of TNFR2-overexpressing keratinocytes (Figure 3).

### 3.4. TRAF2 Inhibition Abrogates the Effect of TWEAK on Keratinocytes

**Figure 4:**
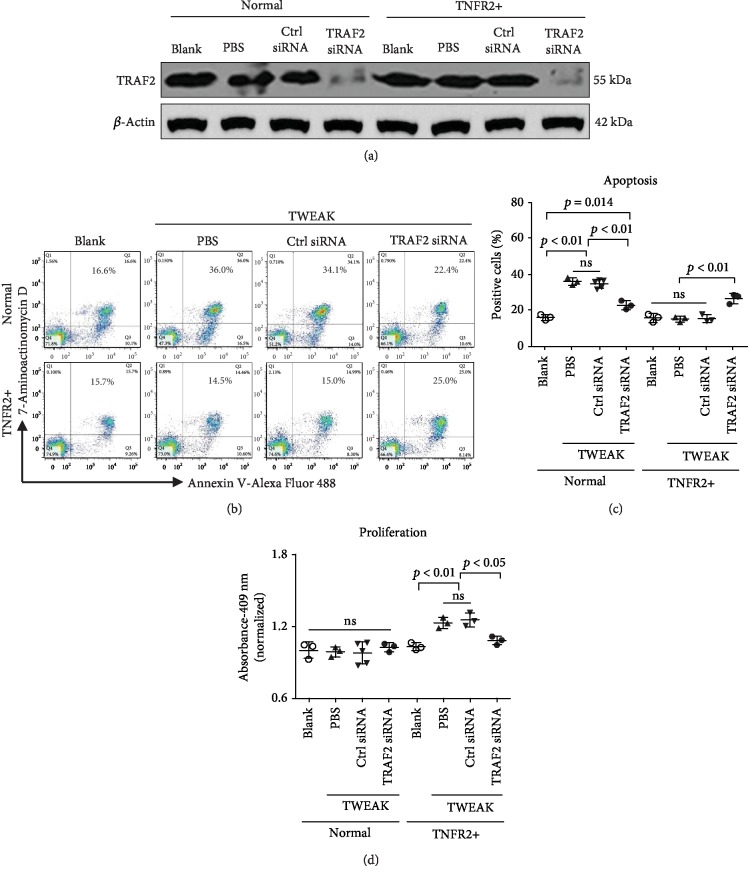
The effect of TRAF2 inhibition on TWEAK-induced cell fates of keratinocytes. Human primary keratinocytes were cultured *in vitro* and received 48 h stimulation with TWEAK (100 ng/ml). TNFR2-overexpressing keratinocytes were constructed using a retroviral vector. Some cells were pretransfected with siRNA against TRAF2 or a control target. (a) TRAF2 protein was detected by Western blotting. (b) Apoptotic cells were quantified by flow cytometry. (c) The ratios of apoptotic cells were compared between different groups. (d) The proliferation of keratinocytes was quantitated by spectrophotometry, followed by comparison across groups. Data were obtained from five independent experiments. Representative images are shown. ns: not significant.

Since TRAF2 exhibits binding affinities with all other proteins we measured (Fn14, cIAP1, TNFR1, and TNFR2), we hypothesized that it may transduce signals from Fn14 activation to the other molecules. Therefore, we next studied the effect of TWEAK in keratinocytes whose expression of TRAF2 was knocked down via siRNA (Figure 4(a)). We found that TWEAK-induced apoptosis of normal keratinocytes was significantly reduced after knockdown of TRAF2 (Figures 4(b) and 4(c)). We performed a similar study in TNFR2-overexpressing keratinocytes and found that knockdown of TRAF2 via siRNA increased the apoptosis of these keratinocytes upon TWEAK stimulation (Figures 4(b) and 4(c)).

In normal keratinocytes, knockdown of TRAF2 caused no measurable impact on proliferation (Figure 4(d)). However, it did reduce TWEAK's ability to stimulate proliferation in TNFR2-overexpressing keratinocytes (Figure 4(d)).

### 3.5. Interaction of TWEAK and Fn14 Activates TNFR1-Dependent Downstream Signals

**Figure 5:**
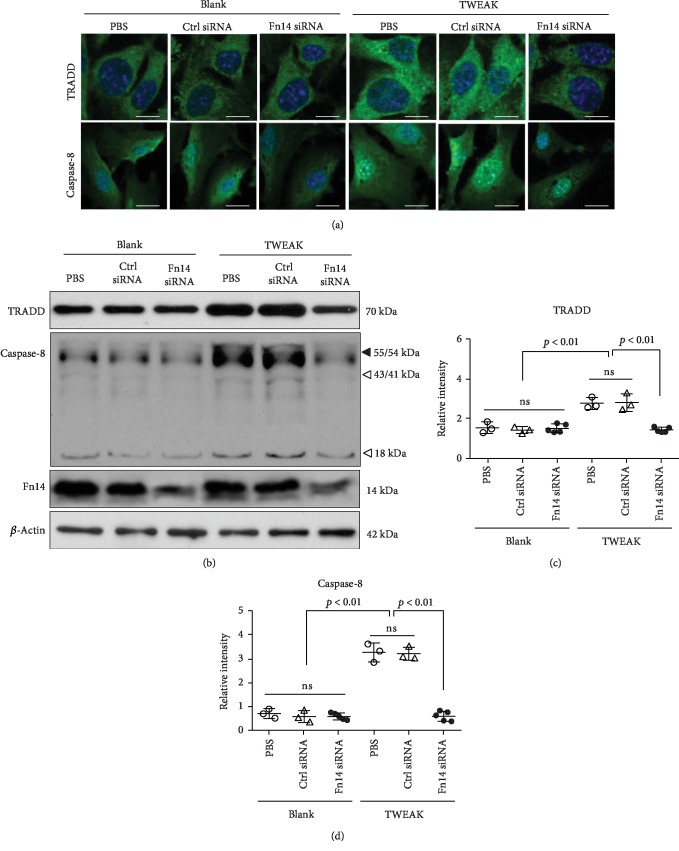
The effect of TWEAK/Fn14 interaction on TNFR1-dependent downstream signals in normal keratinocytes. Primary human keratinocytes were cultured *in vitro* and then received 48 h stimulation with TWEAK (100 ng/ml). Some cells were pretransfected with Fn14 or control siRNA. (a) The expression of TRADD and caspase-8 was analyzed in cells via immunofluorescent staining. (b) The expression of TRADD and caspase-8 was detected in cell lysates via Western blot. (c, d) The band intensities of Western blots were measured with ImageJ software. Data were obtained from five independent experiments. Representative images are shown. Scale bar = 5 *μ*m. ns: not significant.

The effect of TWEAK/Fn14 activation on death signaling pathways was studied in normal keratinocytes that predominantly express TNFR1 [2]. Using immunofluorescence, we found that the expression level of TNFR1-associated death domain protein (TRADD) was increased in TWEAK-stimulated cells and was reduced upon pretreatment with Fn14 siRNA (Figure 5(a)). Similarly, the expression of caspase-8 protein varied in response to TWEAK stimulation or pretreatment with Fn14 siRNA transfection (Figure 5(a)). The level of expression of these two proteins was further characterized by Western blotting. We found increased expression after TWEAK stimulation, but this effect was decreased upon knockdown of Fn14 (Figures 5(b)–5(d)). Fn14 siRNA transfection alone exhibited no effect on the expression of TRADD and caspase-8 (Figure 5).

Importantly, we observed that TWEAK and TNF-*α* exhibited an additive effect on the induction of both caspase-3 and caspase-8 in normal keratinocytes. In good agreement with our prior data, knockdown of Fn14 reduced TWEAK's ability to promote apoptosis of these keratinocytes (Supplementary Fig. [Supplementary-material supplementary-material-1]).

### 3.6. Interaction between TWEAK and Fn14 Promotes Cytoplasmic Import of cIAP1 in TNFR2-Overexpressing Keratinocytes

**Figure 6:**
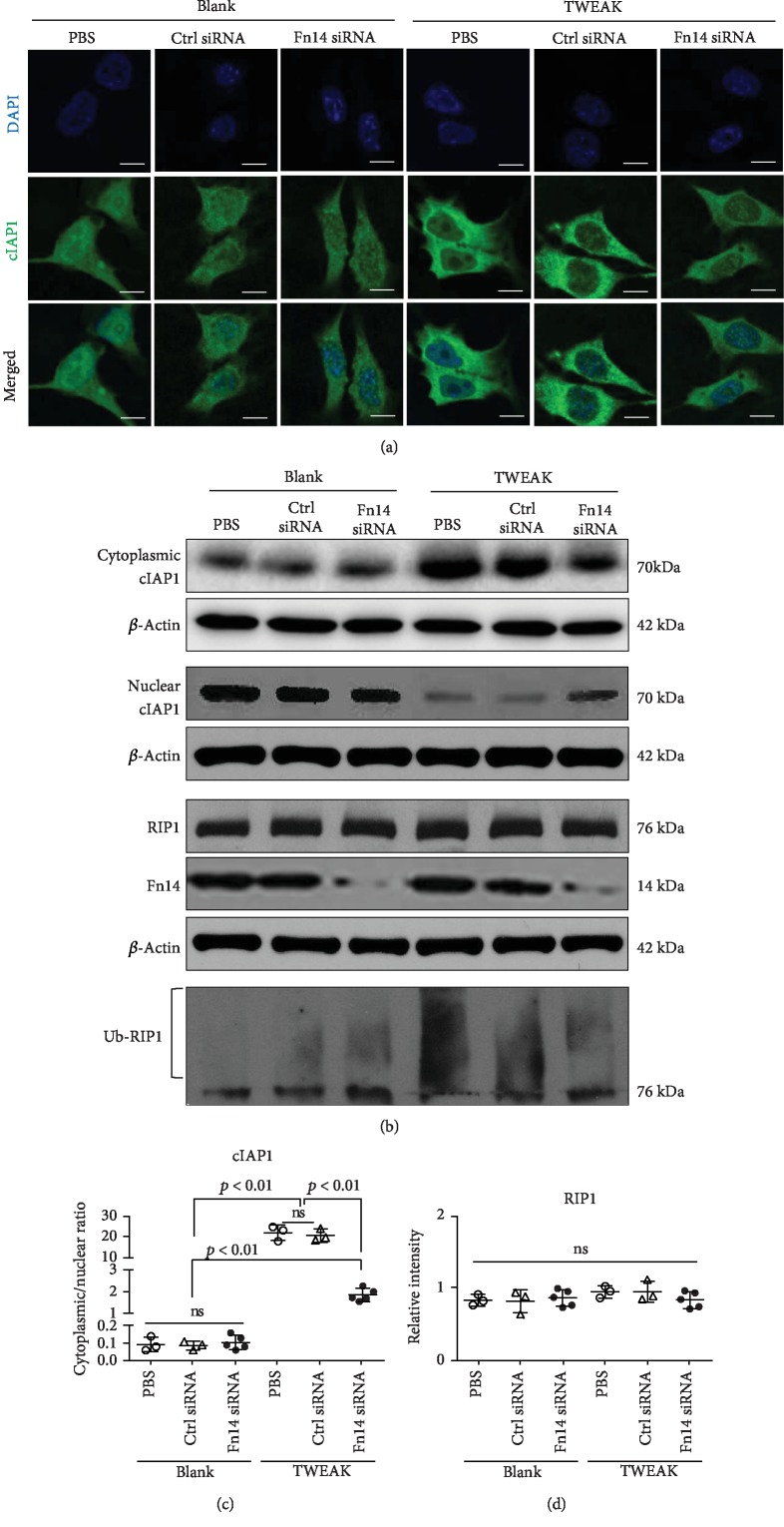
The effect of TWEAK/Fn14 interaction on cytoplasmic import of cIAP1 in TNFR2-overexpressing keratinocytes. Primary human keratinocytes were cultured *in vitro*. TNFR2-overexpressing keratinocytes were constructed using a retroviral vector. Some cells were pretransfected with siRNA against Fn14 or a control target and then received 48 h stimulation with TWEAK (100 ng/ml). (a) The distribution of cIAP1 was analyzed in cells via immunofluorescent staining. (b) The expression of cIAP1 or RIP1 protein was determined in cell lysates or cytoplasmic (or nuclear) fraction via Western blot. In the bottom of this panel, ubiquitinated RIP1 was detected by immunoprecipitation with anti-RIP1 IgG and then antiubiquitin IgG. (c, d) The band intensities of cIAP1 and RIP1 were measured with ImageJ. Data were obtained from five independent experiments. Representative images are shown. Scale bar = 5 *μ*m. ns: not significant.

cIAP1 ubiquitinates receptor-interacting serine/threonine-protein kinase 1 (RIP1) in the cytoplasm, and cIAP1 deletion in the epidermis leads to keratinocyte death [24]. We found that a cIAP1 inhibitor (Birinapant) abrogated TWEAK-induced proliferation in TNFR2-overexpressing keratinocytes (Supplementary Fig. [Supplementary-material supplementary-material-1]), which have been shown to be resistant to death [2]. Next, we investigated the effect of TWEAK on the cIAP1 distribution in these keratinocytes. Using immunofluorescence, we found that TWEAK induced increased cytoplasmic concentration of cIAP1 in these cells (Figure 6(a)). Findings from our Western blot suggested that the cytoplasmic/nuclear ratio of cIAP1 increased with TWEAK stimulation (Figures 6(b) and 6(c)). However, this promotion effect was reduced by knockdown of Fn14 (Figures 6(b) and 6(c)). The expression of RIP1 protein was not affected by either TWEAK stimulation or Fn14 knockdown (Figures 6(b) and 6(d)). Interestingly, TWEAK induced more ubiquitinated RIP1 in these cells, and this effect was partially attenuated upon knockdown of Fn14 (Figure 6(b)). There was no apartment impact of TWEAK on either cIAP1 distribution or RIP1 ubiquitination in normal keratinocytes (Supplementary Fig. [Supplementary-material supplementary-material-1]).

## 4. Discussion

In this study, we demonstrated that a complex containing Fn14/TRAF2/cIAP1/TNFR forms in keratinocytes. Additionally, we found that TRAF2 binds specifically to Fn14, cIAP1, and TNFR. Our results suggest that the expression of TNFR1 versus 2 determines the fate of keratinocytes in the context of TWEAK stimulation and that inhibition of TRAF2 abrogates this effect. Additionally, we found that the TWEAK/Fn14 interaction can enhance the expression of TRADD and caspase-8 in normal keratinocytes and the cytoplasmic import of cIAP1 in TNFR2-overexpressing keratinocytes. Therefore, we believe that signal transduction between Fn14 and TNFR may be a crucial element of TWEAK's regulation of the fate of keratinocytes.

TRAF2 associates with and mediates signal transduction from TNFR. It can directly interact with TNFR and also forms complexes with other TRAF proteins [16, 25]. Previous studies have demonstrated that the TWEAK/Fn14 interaction upregulates the expression of TRAF2 and recruits the cIAP1-TRAF2 complex [2, 15]. Moreover, inhibition of TRAF2 has been shown to reduce TWEAK-facilitated proliferation in keratinocytes [2]. Clearly, TRAF2 plays a key role in the TWEAK/Fn14 signaling pathway. In this work, we confirmed direct associations between TRAF2 and other partners including Fn14, TNFR1, TNFR2, and cIAP1 in keratinocytes, suggesting the formation of a Fn14/TRAF2/cIAP1/TNFR complex. Interestingly, anti-Fn14 IgG only precipitated the other three proteins in TWEAK-stimulated keratinocytes. We interpret this to mean that the TWEAK/Fn14 interaction might the structure of Fn14, thus enabling associations with Fn14.

SPR is a useful tool for the determination of binding kinetics between small molecules [26]. We found that Fn14 binds specifically to TRAF2, but not to cIAP1, TNFR1, or TNFR2. We also found no binding affinity between cIAP1 and TNFR1 or TNFR2; however, TRAF2 exhibits high affinities with all these proteins. These findings provide strong evidence that TRAF2 is a key scaffold molecule in the formation of the Fn14/TRAF2/cIAP1/TNFR complex. We found that siRNA-mediated knockdown of TRAF2 abrogated the TWEAK-induced apoptosis of normal keratinocytes as well as the TWEAK-facilitated proliferation of TNFR2-overexpressing keratinocytes. Therefore, we believe that TRAF2 activation is an intermediate event that does not directly decide the direction of TWEAK's impact on cellular fate.

Instead, it appears that the TNFR expression profile determines the type of effect that TWEAK has on keratinocytes [12]. Similar to TNF-*α* [27], TWEAK induces death in cells that predominantly express TNFR1 but induces cell proliferation in cells that overexpress TNFR2. The common consensus is that TNFR2 is restricted mainly to immune and endothelial cells and also acts to sustain the function of regulatory T cells [27]. However, recent studies have suggested that TNFR2 is also expressed in cancer cells [28], HPV-infected keratinocytes [2], and psoriatic keratinocytes [13], which all possess proliferative properties. Local microenvironments, physical stimuli, or phenotype transformation may influence the expression profile of TNFR [2, 4, 5, 13], leading to alteration of keratinocyte fates upon TWEAK/Fn14 activation. In this study, we verified that TWEAK acts independently or cooperates with TNF-*α* in its role as a regulator of keratinocytes. However, the exact mechanistic interactions between these two cytokines require further investigation.

We observed that TWEAK/Fn14 activation upregulates the expression of TRADD and caspase-8 in normal keratinocytes, which predominantly express TNFR1 and undergo death upon TWEAK stimulation [1, 14]. TRADD is a TNFR1 partner with a death domain that recruits Fas-associated protein along with DD and caspase-8 to trigger apoptotic signals [29]. Moreover, depletion of Fas-associated protein with DD or caspase-8 can attenuate TWEAK-induced cell death [30]. In the context of TNFR1 signaling, priming of tumor cells with TWEAK enhances TNF-induced caspase-8 activation and apoptosis [25]. Hence, TWEAK triggers TNFR1-mediated death of cells in a TRADD- and caspase-8-dependent manner.

More importantly, we found that the TWEAK/Fn14 interaction promotes cytoplasmic import of cIAP1 in TNFR2-overexpressing keratinocytes. We also found that TWEAK induces ubiquitination of RIP1 in these cells. It is well known that cIAP1 ubiquitinates RIP1 in cytoplasm and that cIAP1 deletion in the epidermis leads to death of keratinocytes [24]. cIAP1 is required for TNFR2-induced canonical NF-*κ*B activation and is recruited to the TNFR2 signaling complex upon TNF-*α* stimulation [31]. In fact, TNFR2-induced canonical NF-*κ*B activation has been demonstrated to correlate with the proliferation of cells [32]. Thus, TWEAK promotes the TNFR2-related proliferation of keratinocytes possibly through a mechanism involving the cytoplasmic import of cIAP1 and RIP1 ubiquitination. There was no apparent effect of TWEAK on either cIAP1 distribution or RIP1 ubiquitination in normal keratinocytes. This may be due to a generally lower level of TNFR2 expression in normal keratinocytes.

**Figure 7:**
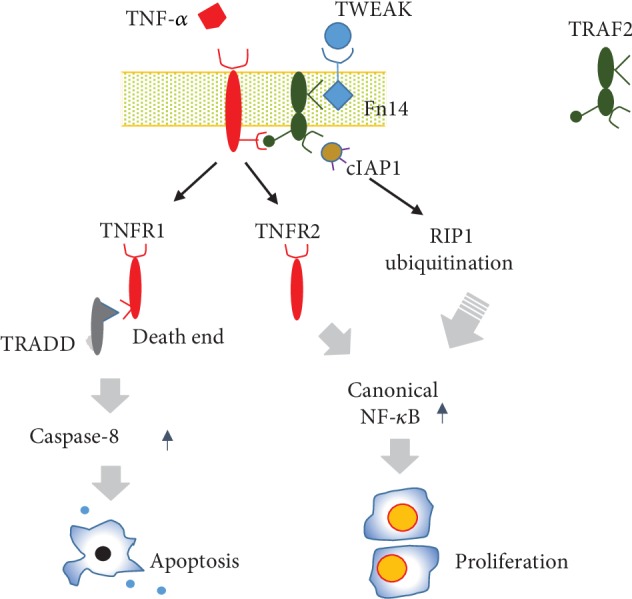
Diagram of the function of the Fn14-TRAF2-TNFR signaling axis. TWEAK/Fn14 interaction acts independently or cooperates with TNF-*α* in triggering TNFR-mediated signals. In the TNFR1-predominant cells (such as normal keratinocytes), TWEAK induces cell death through activation of the TRADD and caspase-8 pathways. Conversely, TWEAK induces proliferation of cells that overexpress TNFR2 (such as HPV-infected keratinocytes). The Fn14-TRAF2-TNFR axis is central to TWEAK's regulation of cell fate.

In conclusion, TWEAK triggers the formation of a Fn14/TRAF2/cIAP1/TNFR complex in keratinocytes. In the context of TWEAK stimulation, the TNFR expression profile largely determines whether keratinocytes will proliferate or undergo apoptosis. Moreover, TWEAK induces the death of normal keratinocytes by activating the TRADD and caspase-8 pathways and triggers the proliferation of TNFR2-overexpressing keratinocytes alongside increased cytoplasmic import of cIAP1 (Figure 7). Therefore, an Fn14/TRAF2/TNFR signaling axis mediates TWEAK's capacity to regulate the fate of keratinocytes. Future studies should explore the structural and functional changes of these binding partners during TWEAK stimulation, investigate the potential therapeutic implications of this axis in inflammatory and immune-mediated skin diseases.

## Data Availability

The data used to support the findings of this study are included within the article and the supplementary information file.

## Abbreviations

cIAP1: Cellular inhibitor of apoptosis protein 1
Fn14: Fibroblast growth factor-inducible 14
HPV: Human papillomavirus
qRT-PCR: Quantitative real-time polymerase chain reaction
SPR: Surface plasmon resonance
TNF: Tumor necrosis factor
TNFR: TNF receptor
TRADD: TNFR1-associated death domain protein
TRAF: TNFR-associated factor
TWEAK: TNF-like weak inducer of apoptosis.

## Supplementary Materials

Supplementary Materials Figure S1: the potential effect of TWEAK on autocrine TNF-*α* production. Human primary keratinocytes were cultured in vitro. Some cells were treated with bovine serum albumin or TWEAK (100 ng/ml, 48 h). (a) The mRNA expression levels of TNF-*α* were determined by qRT-PCR. (b) By using an enzyme-linked immunosorbent assay, TNF-*α* protein was determined in supernatants. Data were obtained from three independent experiments. ns: not significant. Figure S2: the effect of siRNA transfection on cytokine production. Human primary keratinocytes were cultured in vitro and received the transfection of control or target siRNA. The mRNA expression levels of TNF-*α*, RANTES, and interferon *γ* were determined by qRT-PCR. (a) Cells were transfected with control or Fn14 siRNA. (b) Cells were transfected with control or TRAF2 siRNA. Data were obtained from three experiments. ns: not significant. Figure S3: the efficiencies of siRNA transfection and retroviral infection in human primary keratinocytes. Cells were cultured in vitro and received the transfection of control or target siRNA, or the infection of vector that was inserted with target gene or not. (a) The mRNA expression levels of Fn14 were determined accordingly. (b) By Western blotting, Fn14 protein was determined in lysates. Similarly, the mRNA and protein expression levels of TRAF2 (c, d) and TNFR2 (e, f) were determined in cells. Data were obtained from three experiments. Representative images are shown. ns: not significant. Figure S4: the expressions of markers in nuclear and cytoplasmic fractions of cells. Human keratinocytes were cultured in vitro, then processed for nuclear or cytoplasmic fraction. The markers of nuclear (MSH2) and cytoplasmic (HSP90) proteins were detected by Western blotting. (a) The markers were detected in fractions of primary keratinocytes. (b) The markers were detected in fractions of TNFR2-overexpressing keratinocytes. Data were obtained from three independent experiments. Representative images are shown. MSH2: MutS protein homolog 2. HSP90: heat shock protein 90. Figure S5: the validation of anti-cIAP1 IgG. (a) Protein extracts were precipitated by using rabbit anti-cIAP1 IgG (clone # ab108361) or control IgG (clone # ab172730). The pellets were resuspended for gel analysis and Coomassie blue staining. (b) The bands at 70 kDa were verified by Western blotting with anti-cIAP1 IgG as primary antibody. (c) Proteins were extracted from the bands at 70 kDa and then processed for MALDI-TOF mass spectrometry analysis. Figure S6: the effect of TWEAK stimulation on TNFR expression in keratinocytes. Human primary keratinocytes were cultured in vitro. Some cells were treated with TWEAK (0-100 ng/ml, 24 h). (a) By qRT-PCR, the mRNA expression levels of TNFR1 were determined. (b) By Western blotting, TNFR1 protein was determined in lysates. The intensities of blot bands were measured with ImageJ software. Similarly, the mRNA and protein expression levels of TNFR2 (c, d) were determined in cells. Data were obtained from three independent experiments. Representative images are shown. Figure S7: the specific affinities between the Fn14, TRAF2, TNFR1, TNFR2, and cIAP1 molecules. The recombinant proteins were analyzed by SPR. Fn14 protein was immobilized to sensor chip, followed by running TRAF2 (a), cIAP1 (b), TNFR1 (c), or TNFR2 (d). cIAP1 protein was immobilized to sensor chip, followed by running TNFR1 (e) or TNFR2 (f). The running samples were prepared at a concentration rage of 0–100 nM. Data were obtained from three to five independent experiments. Representative images are shown. Figure S8: the effect of TWEAK on apoptosis of keratinocytes. Primary keratinocytes were cultured in vitro, then received 48 h stimulation of TWEAK (100 ng/ml) or TNF-*α* (10 ng/ml). Some cells were pretransfected with control or Fn14 siRNA. (a) By Western blotting, the caspase-3 and caspase-8 proteins were detected in cell lysates. (b) The band intensities of Western blots were measured with ImageJ software. (c) The apoptotic cells were determined by flow cytometry. (d) The ratios of apoptotic cells were compared accordingly. Data were obtained from five independent experiments. Representative images are shown. ^∗^*p* < 0.05, compared with the blank group; ^#^*p* < 0.05, compared with the TWEAK-alone group; ^∆^*p* < 0.05, compared with the TNF-*α* alone (b) or control siRNA (d) group. Figure S9: the effect of cIAP1 inhibitor on TWEAK-induced proliferation of keratinocytes. TNFR2-overexpressing keratinocytes were cultured in vitro, then received 48 h stimulation of TWEAK (100 ng/ml) or Birinapant (1 *μ*M). (a) The apoptotic cells were determined by flow cytometry. (b) The ratios of apoptotic cells were compared accordingly. (c) The proliferation of keratinocytes was quantitated and then compared between different groups. Data were obtained from three to five independent experiments. Representative images are shown. ^∗^*p* < 0.05, compared with the blank group; ^#^*p* < 0.05, compared with the Birinapant group; ^∆^*p* < 0.05, compared with the TWEAK group. Figure S10: the effect of TWEAK/Fn14 interaction on the cytoplasmic import of cIAP1 in normal keratinocytes. Human primary keratinocytes were cultured in vitro, then received 48 h stimulation of TWEAK (100 ng/ml). Some cells were pretransfected with Fn14 or control siRNA. (a) By immunofluorescence, the distribution of cIAP1 was analyzed in cells. (b) By Western blotting, the cIAP1 or RIP1 protein was determined in cell lysates or cytoplasmic (or nuclear) fraction. In the bottom of this panel, ubiquitinated RIP1 was detected by immunoprecipitation with anti-RIP1 IgG and then probation with antiubiquitin IgG. (c, d) The band intensities of cIAP1 and RIP1 were measured with ImageJ software. Data were obtained from three to five independent experiments. Representative images are shown. ns: not significant.
