# Supplementary material for: Tumor Necrosis Factor (TNF) Receptor Expression Determines Keratinocyte Fate upon Stimulation with TNF-Like Weak Inducer of Apoptosis
Source: Mediators Inflamm. 2019 Dec 5;2019:2945083. doi: 10.1155/2019/2945083 (PMC6915140; doi:10.1155/2019/2945083)
Supplement: Supplementary Materials — Figure S1: the potential effect of TWEAK on autocrine TNF-α production. Human primary keratinocytes were cultured in vitro. Some cells were treated with bovine serum albumin or TWEAK (100 ng/ml, 48 h). (a) The mRNA expression levels of TNF-α were determined by qRT-PCR. (b) By using an enzyme-linked immunosorbent assay, TNF-α protein was determined in supernatants. Data were obtained from three independent experiments. ns: not significant. Figure S2: the effect of siRNA transfection on cytokine production. Human primary keratinocytes were cultured in vitro and received the transfection of control or target siRNA. The mRNA expression levels of TNF-α, RANTES, and interferon γ were determined by qRT-PCR. (a) Cells were transfected with control or Fn14 siRNA. (b) Cells were transfected with control or TRAF2 siRNA. Data were obtained from three experiments. ns: not significant. Figure S3: the efficiencies of siRNA transfection and retroviral infection in human primary keratinocytes. Cells were cultured in vitro and received the transfection of control or target siRNA, or the infection of vector that was inserted with target gene or not. (a) The mRNA expression levels of Fn14 were determined accordingly. (b) By Western blotting, Fn14 protein was determined in lysates. Similarly, the mRNA and protein expression levels of TRAF2 (c, d) and TNFR2 (e, f) were determined in cells. Data were obtained from three experiments. Representative images are shown. ns: not significant. Figure S4: the expressions of markers in nuclear and cytoplasmic fractions of cells. Human keratinocytes were cultured in vitro, then processed for nuclear or cytoplasmic fraction. The markers of nuclear (MSH2) and cytoplasmic (HSP90) proteins were detected by Western blotting. (a) The markers were detected in fractions of primary keratinocytes. (b) The markers were detected in fractions of TNFR2-overexpressing keratinocytes. Data were obtained from three independent experiments. Representative images are s [file 2945083.f1.pdf]

Supplementary material

Tumor necrosis factor (TNF) receptor expression determines keratinocyte fate upon stimulation with TNF-like weak inducer of apoptosis

Xuening Wang[1] • Dan Cheng[2] • Guanglei Hu[1] • Lili Liang[1] • Fei Tan[1] • Tong Xiao[1] • Shengxiang Xiao[1] • Yumin Xia[1]

[1]Department of Dermatology, The Second Affiliated Hospital, School of Medicine, Xi'an Jiaotong University, Xi'an, China

[2]College of Life Sciences, Wuhan University, Wuhan, China

Correspondence: Yumin Xia, Department of Dermatology, The Second Affiliated Hospital of Xi'an Jiaotong University, 157 Xiwu Road, Xi'an 710004, China. Email: xiayumin1202@163.com, Tel: +86-29-87678324, Fax: +86-29-87678324

Table S1. Primer sets used for sequencing expression genes.

Gene	Primer (5'-3')
Human Fn14	F: CTCTGAGCCTGACCTTCGTG<br>R: GGGGGCACATTGTCACTGGA
Human TNF-α	F: CTATCTGGGAGGGGTCTTCC<br>R: GGTTGAGGGTGTCTGAAGGA
Human TNFR2	F: AGCTCCACAATGGGAGACAC<br>R: TGGTTAACTGGGCTTCATCC
Human TRAF2	F: AAGACGGCCACTTTTGAGAA<br>R: CCAAGACCTTCTGCTCCAAG
Human GAPDH	F: GCACCGTCAAGGCTGAGAAC<br>R: TGGTGAAGACGCCAGTGGA

Abbreviations: Fn14, fibroblast growth factor-inducible 14; TNF, tumor necrosis factor; TNFR, tumor necrosis factor receptor; TRAF, TNFR-associated factor; GAPDH, glyceraldehyde 3-phosphate dehydrogenase; F: forward; R: reverse.

Table S2. SPR analysis of binding affinities.

Immobilization	Injection	MES buffer	K_a (10^4/Ms)	K_d (10^{-5}/s)	K_D (10^{-9} M)	Rmax (RU)
TRAF2	Fn14	pH 6.109	0.82±0.11	26.40±0.86	32.20±0.39	103
TRAF2	TNFR1	pH 6.109	5.37±0.40	35.07±0.25	6.53±0.17	191
TRAF2	TNFR2	pH 6.109	6.68±0.18	16.10±0.22	2.41±0.26	182
TRAF2	cIAP1	pH 6.109	3.41±0.12	3.21±0.24	0.94±0.19	140
Fn14	cIAP1	pH 5.820	/	/	/	/
Fn14	TNFR1	pH 5.820	/	/	/	/
Fn14	TNFR2	pH 5.820	/	/	/	/
cIAP1	TNFR1	pH 5.725	/	/	/	/
cIAP1	TNFR2	pH 5.725	/	/	/	/

Abbreviations: cIAP1, cellular inhibitor of apoptosis protein 1; Fn14, fibroblast growth factor-inducible 14; MES, 2-(N-morpholino)ethanesulfonic acid; RU, response unit; SPR, surface plasmon resonance; TNFR, tumor necrosis factor receptor; TRAF2, tumor necrosis factor-associated factor 2.

Notes: Proteins were immobilized to chip at a concentration of 10 nM. Rmax meant the highest values at the range of 0 to 120 nM. KD = Kd/Ka.

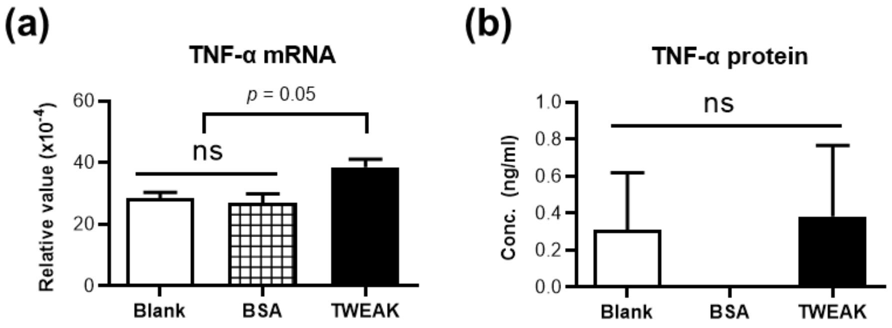

Figure S1. The potential effect of TWEAK on autocrine TNF-α production. Human primary keratinocytes were cultured *in vitro*. Some cells were treated with bovine serum albumin or TWEAK (100 ng/ml, 48 h). **a** The mRNA expression levels of TNF-α were determined by qRT-PCR. **b** By using an enzyme-linked immunosorbent assay, TNF-α protein was determined in supernatants. Data were obtained from three independent experiments. ns, not significant.

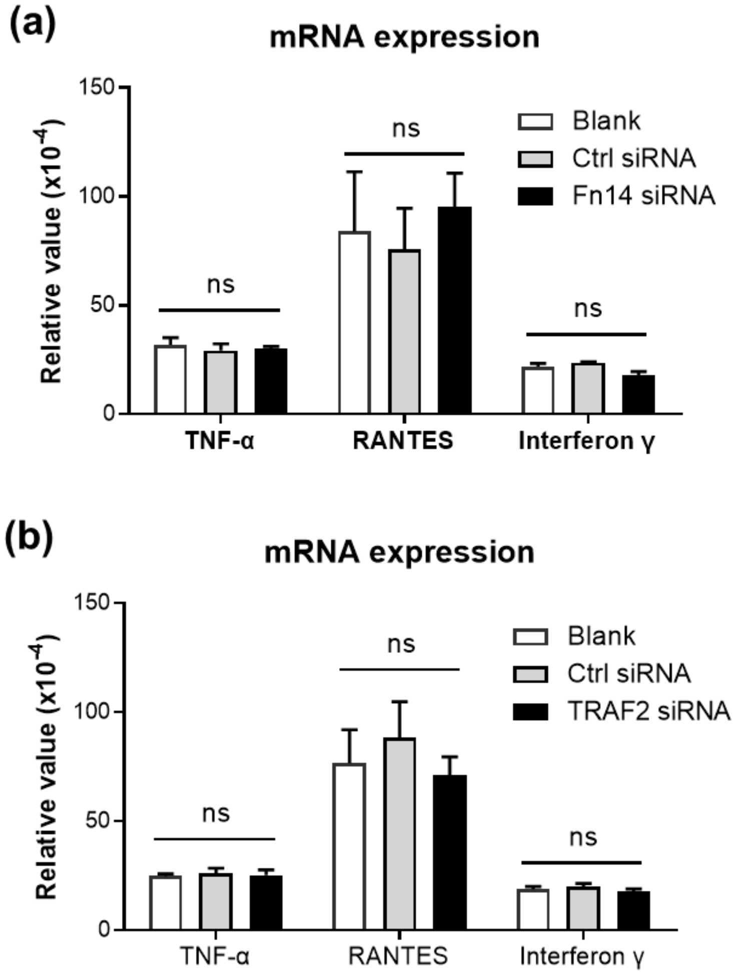

Figure S2. The effect of siRNA transfection on cytokine production. Human primary keratinocytes were cultured *in vitro*, and received the transfection of control or target siRNA. The mRNA expression levels of TNF-α, RANTES, and interferon γ were determined by qRT-PCR. **a** Cells were transfected with control or Fn14 siRNA. **b** Cells were transfected with control or TRAF2 siRNA. Data were obtained from three experiments. ns, not significant.

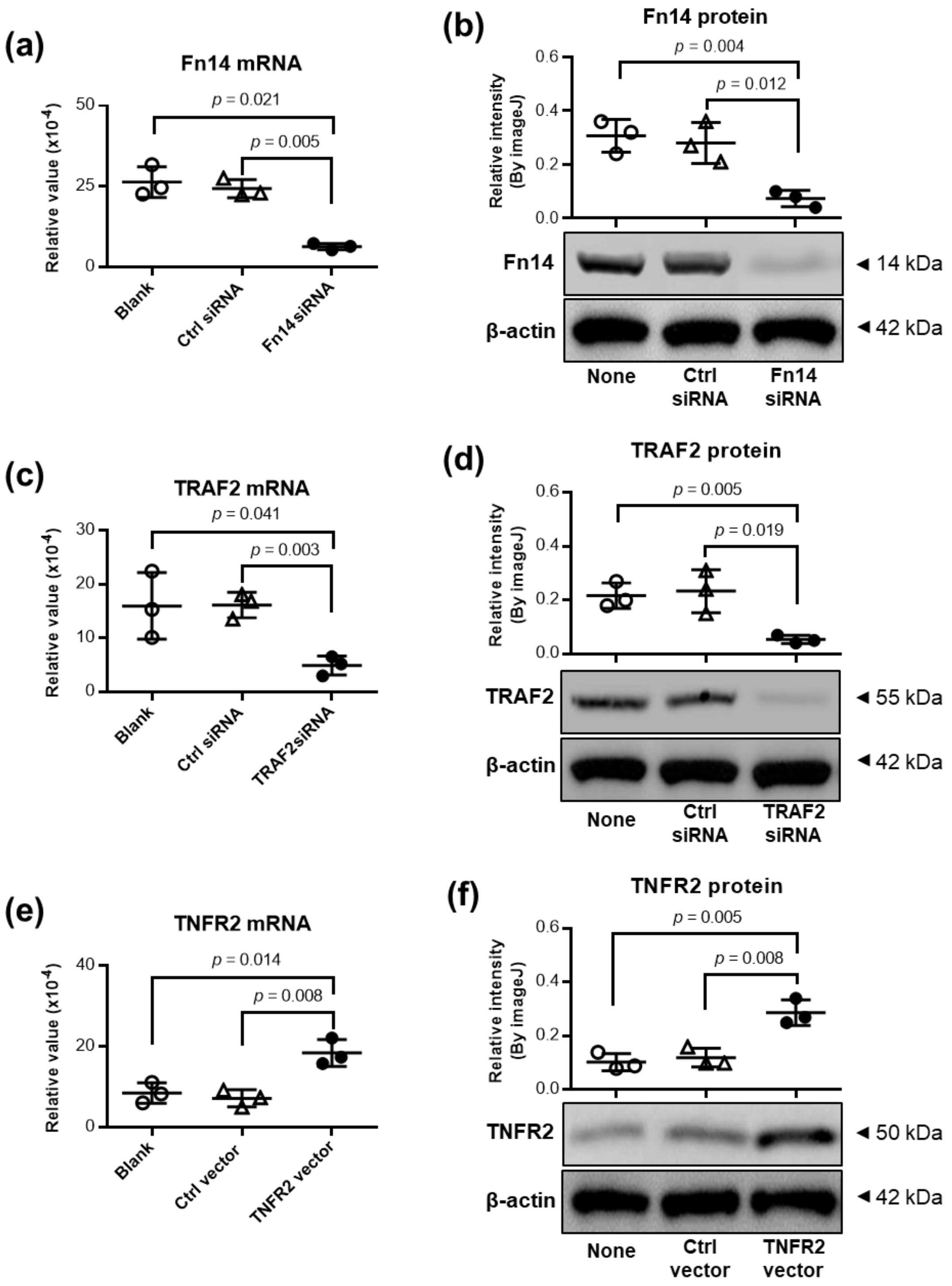

Figure S3. The efficiencies of siRNA transfection and retroviral infection in human primary keratinocytes. Cells were cultured *in vitro*, and received the transfection of control or target siRNA, or the infection of vector that was inserted with target gene or not. **a** The mRNA expression levels of Fn14 were determined accordingly. **b** By

Western blotting, Fn14 protein was determined in lysates. Similarly, the mRNA and protein expression levels of TRAF2 (**c**, **d**) and TNFR2 (**e**, **f**) were determined in cells. Data were obtained from three experiments. Representative images are shown. ns, not significant.

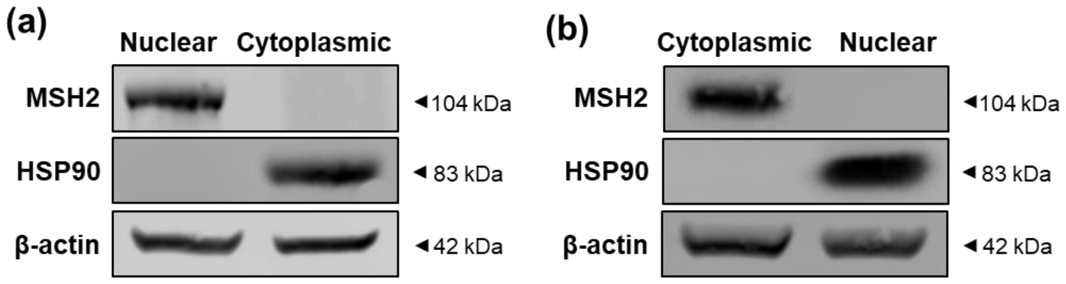

Figure S4. The expressions of markers in nuclear and cytoplasmic fractions of cells. Human keratinocytes were cultured *in vitro*, then processed for nuclear or cytoplasmic fraction. The markers of nuclear (MSH2) and cytoplasmic (HSP90) proteins were detected by Western blotting. **a** The markers were detected in fractions of primary keratinocytes. **b** The markers were detected in fractions of TNFR2-overexpressing keratinocytes. Data were obtained from three independent experiments. Representative images are shown. MSH2, MutS protein homolog 2. HSP90, heat shock protein 90.

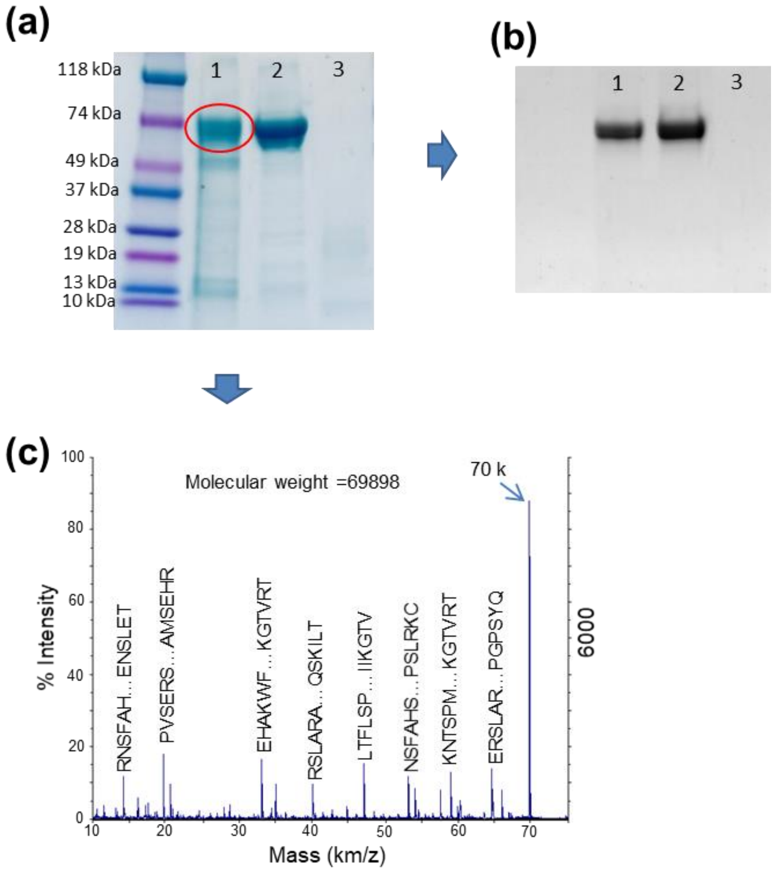

Figure S5. The validation of anti-cIAP1 IgG. **a** Protein extracts were precipitated by using rabbit anti-cIAP1 IgG (clone # ab108361) or control IgG (clone # ab172730). The pellets were re-suspended for gel analysis and Coomassie blue staining. **b** The bands at 70 kDa were verified by Western blotting with anti-cIAP1 IgG as primary antibody. **c** Proteins were extracted from the bands at 70 kDa, and then processed for MALDI-TOF mass spectrometry analysis.

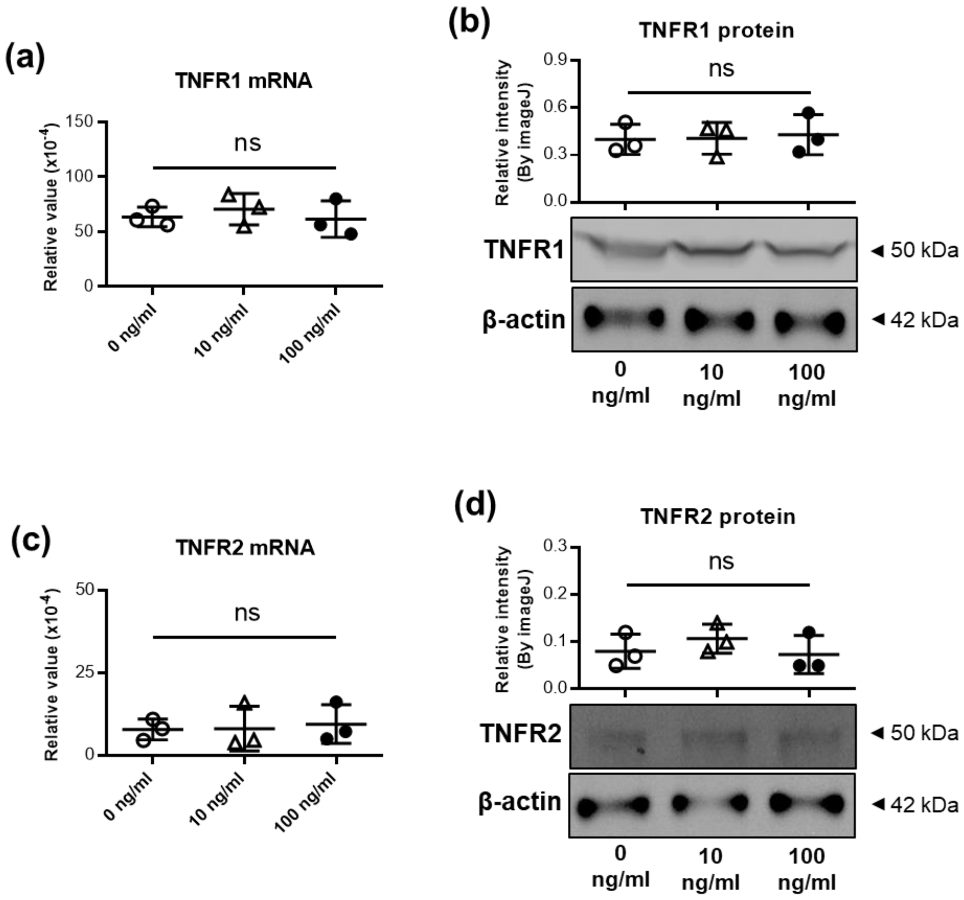

Figure S6. The effect of TWEAK stimulation on TNFR expression in keratinocytes. Human primary keratinocytes were cultured in vitro. Some cells were treated with TWEAK (0-100 ng/ml, 24 h). **a** By qRT-PCR, the mRNA expression levels of TNFR1 were determined. **b** By Western blotting, TNFR1 protein was determined in lysates. The intensities of blot bands were measured with ImageJ software. Similarly, the mRNA and protein expression levels of TNFR2 (**c**, **d**) were determined in cells. Data were obtained from three independent experiments. Representative images are shown.

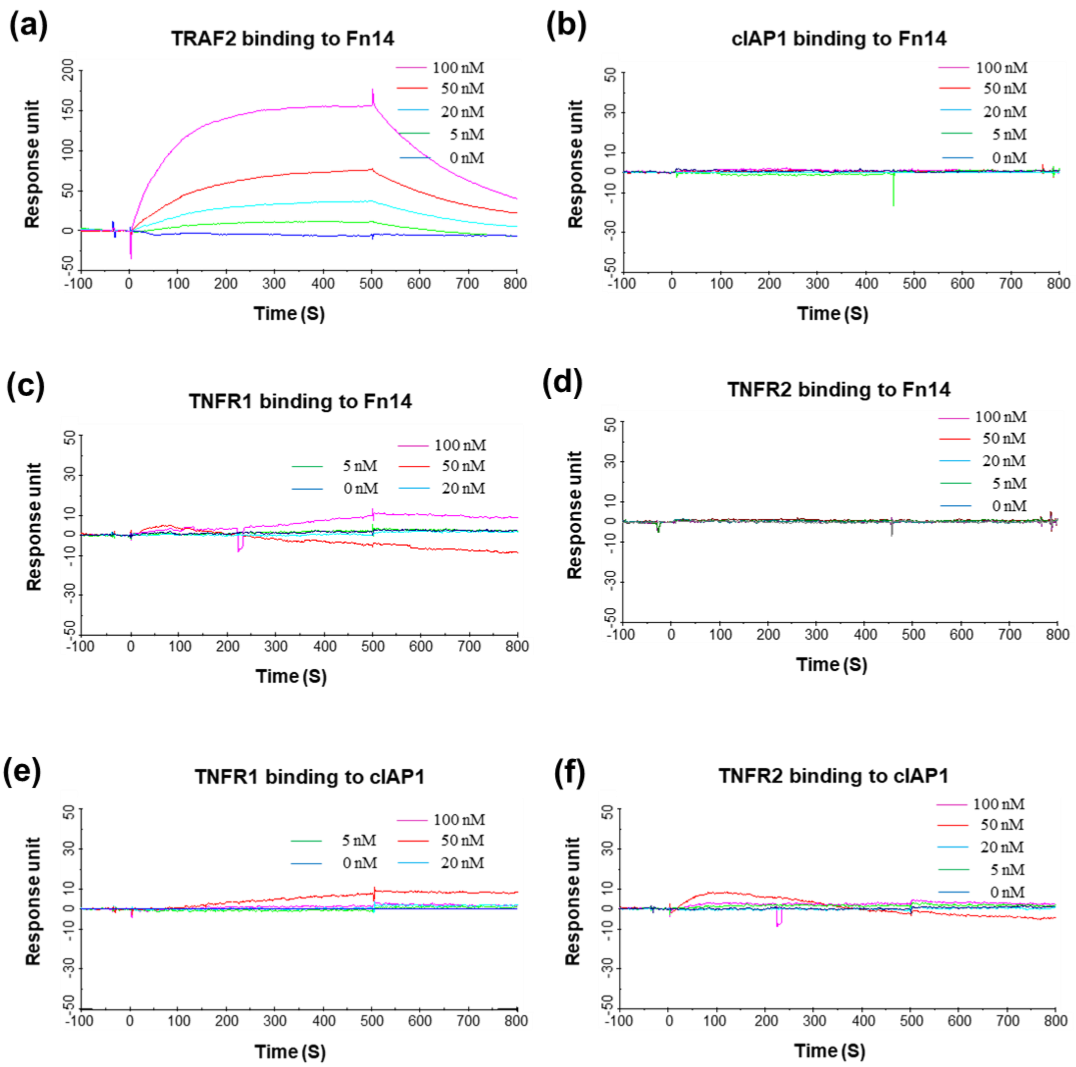

Figure S7. The specific affinities between the Fn14, TRAF2, TNFR1, TNFR2, and cIAP1 molecules. The recombinant proteins were analyzed by SPR. Fn14 protein was immobilized to sensor chip, followed by running TRAF2 (**a**), cIAP1 (**b**), TNFR1 (**c**) or TNFR2 (**d**). cIAP1 protein was immobilized to sensor chip, followed by running TNFR1 (**e**) or TNFR2 (**f**). The running samples were prepared at a concentration rage of 0–100 nM. Data were obtained from three to five independent experiments. Representative images are shown.

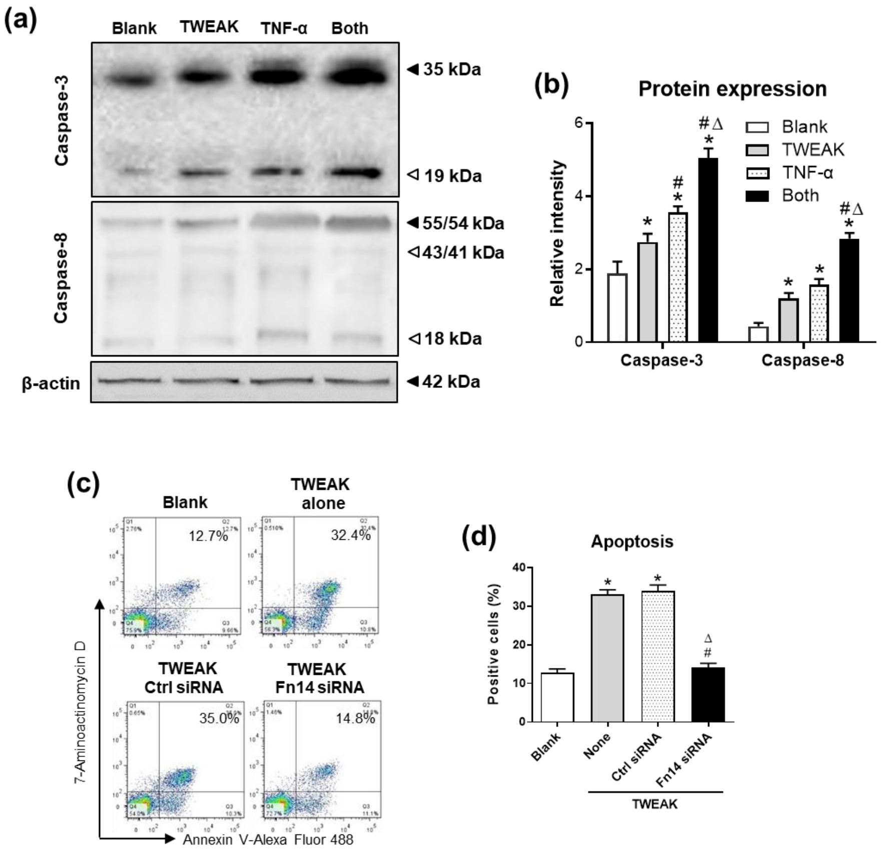

Figure S8. The effect of TWEAK on apoptosis of keratinocytes. Primary keratinocytes were cultured in vitro, then received 48-h stimulation of TWEAK (100 ng/ml) or TNF-α (10 ng/ml). Some cells were pre-transfected with control or Fn14 siRNA. **a** By Western blotting, the caspase-3 and caspase-8 proteins were detected in cell lysates. **b** The band intensities of Western blots were measured with ImageJ software. **c** The apoptotic cells were determined by flow cytometry. **d** The ratios of apoptotic cells were compared accordingly. Data were obtained from five independent experiments. Representative images are shown. *$p < 0.05$, compared with blank group; #$p < 0.05$, compared with TWEAK alone group; Δ$p < 0.05$, compared with TNF-α alone (**b**) or control siRNA (**d**) group.

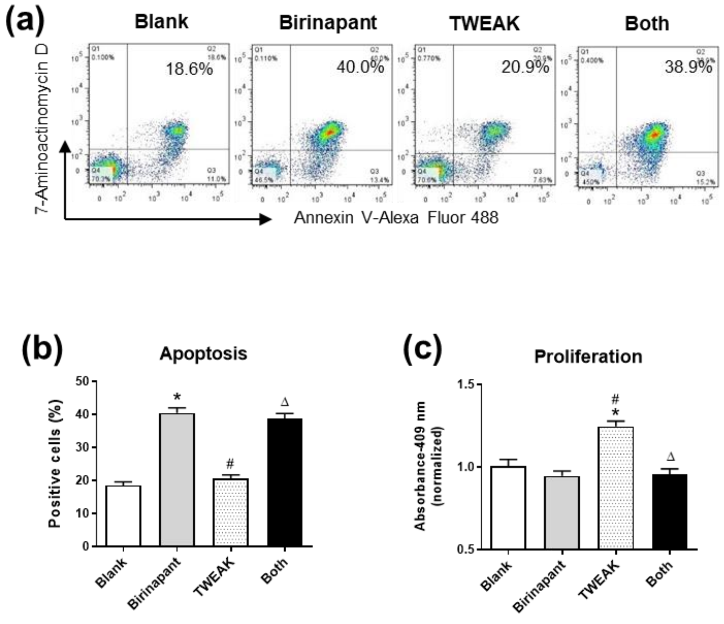

Figure S9. The effect of cIAP1 inhibitor on TWEAK-induced proliferation of keratinocytes. TNFR2-overexpressing keratinocytes were cultured *in vitro*, then received 48-h stimulation of TWEAK (100 ng/ml) or Birinapant (1 μM). **a** The apoptotic cells were determined by flow cytometry. **b** The ratios of apoptotic cells were compared accordingly. **c** The proliferation of keratinocytes was quantitated and then compared between different groups. Data were obtained from three to five independent experiments. Representative images are shown. *$p < 0.05$, compared with blank group; #$p < 0.05$, compared with Birinapant group; Δ$p < 0.05$, compared with TWEAK group.

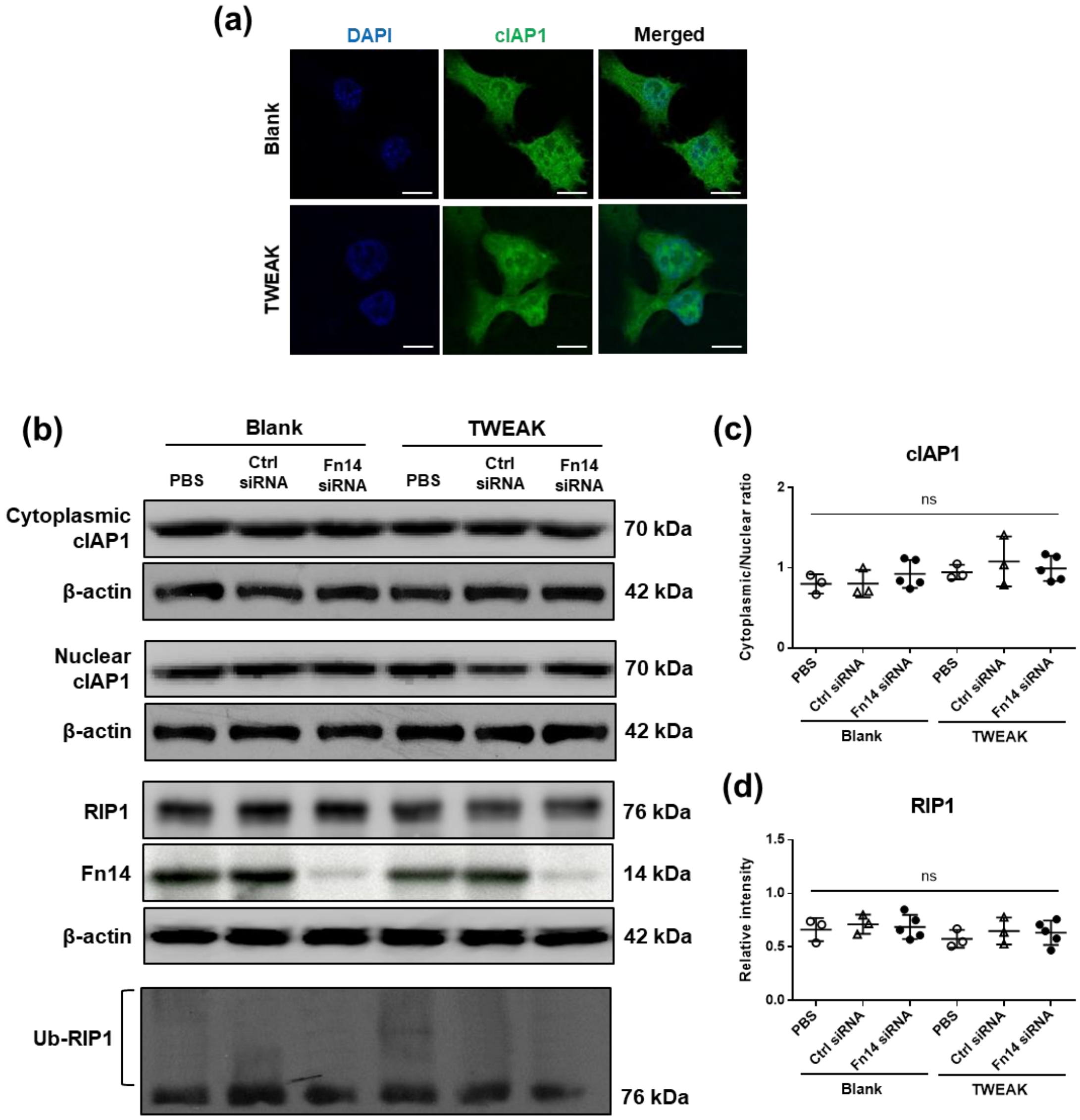

Figure S10. The effect of TWEAK/Fn14 interaction on cytoplasmic import of cIAP1 in normal keratinocytes. Human primary keratinocytes were cultured in vitro, then received 48-h stimulation of TWEAK (100 ng/ml). Some cells were pretransfected with Fn14 or control siRNA. **a** By immunofluorescence, the distribution of cIAP1 was analyzed in cells. **b** By Western blotting, the cIAP1 or RIP1 protein was determined in cell lysates or cytoplasmic (or nuclear) fraction. In the bottom of this panel, ubiquitinated RIP1 was detected by immunoprecipitation with anti-RIP1 IgG and then probation with anti-ubiquitin IgG. **c**, **d** The band intensities of cIAP1 and RIP1 were

measured with ImageJ software. Data were obtained from three to five independent experiments. Representative images are shown. ns, not significant.
